# Role of the erythropoietin receptor in Lung Cancer cells: erythropoietin exhibits angiogenic potential

**DOI:** 10.7150/jca.36924

**Published:** 2020-08-21

**Authors:** Xiaoqing Liu, Amanda Tufman, Juergen Behr, Rosemarie Kiefl, Torsten Goldmann, Rudolf M. Huber

**Affiliations:** 1 Department of Internal Medicine V, Division of Respiratory Medicine and Thoracic Oncology, Ludwig-Maximilians University (LMU), Thoracic Oncology Centre Munich. Comprehensive Pneumology Center Munich, Member of the German Center for Lung Research (DZL), Munich, Bavaria, Germany.; 2 Pathology Department of the University Medical Center Schleswig-Holstein (UKSH), Lübeck Campus and the Borstel Research Center, Lübeck and Borstel, Germany; Airway Research Center North, Member of the German Center for Lung Research (DZL).

**Keywords:** erythropoietin, erythropoietin receptor, lung cancer cells, angiogenesis, *in vivo*

## Abstract

**Background:** Recombinant human erythropoietin (rHuEPO), a hormone regulating the proliferation and differentiation of erythroid cells, is one of the prescription drugs used to treat cancer-associated anemia. However, administration of rHuEPO to cancer patients has been reported to be associated with decreased survival, and the mechanism by which it acts remains controversial. The present study aimed to investigate the expression of the EPO-receptor in lung cancer cell lines and whether rHuEPO treatment affected its growth and migration. Moreover, the angiogenic effects of rHuEPO were also explored *in vivo*.

**Methods:** Expression of the EPO-receptor in lung cancer cell lines was measured by Western blotting and enzyme linked immunosorbent assays (ELISAs). Proliferation of the lung cancer cells was monitored in the presence of rHuEPO. Human umbilical vein endothelial cells (HUVECs) were used for tube formation assays *in vitro*, and transwell migration assays were performed to detect migration under rHuEPO treatment. Matrigel plug technology was employed to observe the angiogenic effects in both nude mice and Matrigel-containing lung cancer cell lines H838 or H1975. Microvessel density (MVD) was measured using CD31 Immunohistochemistry (IHC) staining.

**Results:** EPO-receptor (EPO-R) was only detected in the cell lines H838 and H1339 by ELISA. However, the EPO-R protein was detected in all cell lines by Western blotting, which is in contradiction to the ELISA results. Proliferation and migration were not affected by rHuEPO treatment. However, rHuEPO promoted HUVEC tube formation *in vitro* and significantly induced the formation of new blood vessels *in vivo*. Furthermore, rHuEPO did not antagonize the inhibitory effects of Afatinib (epidermal growth factor receptor-tyrosine kinase inhibitor; EGFR-TKI) in simultaneous treatment with rHuEPO. In a 3D cell co-culture model, rHuEPO did not enhance the secretion of vascular endothelial growth factor (VEGF) in lung cancer cells or human lung fibroblast cell line MRC-5.

**Conclusions:** We have shown that the role of EPO goes beyond erythropoiesis, also playing a strong role in angiogenesis by participating in new blood vessel formation in lung cancer models. Thus, rHuEPO may raise the risk of thrombosis and metastasis *in vivo*. Additionally, our results suggest that studies using commercially available EPO-R antibodies should be reexamined; some of these antibodies may not in fact recognize EPO-R.

## Introduction

Erythropoietin (EPO), also known as hematopoietin or hemopoietin, is a 30.4-kDa glycoprotein that induces erythropoiesis and is produced mainly by the kidneys and the liver in response to hypoxia 1. It is a protein signaling molecule (cytokine) that acts on red blood cell precursors. EPO binds to the erythropoietin receptor (EPO-R), which is encoded by the EPOR gene 2.

EPO-R is a membrane-bound receptor present in erythroblasts and is a member of the cytokine super family of type 1 transmembrane proteins 3. The calculated size of the EPO-R protein is 52.6 kDa 4. EPO-R is expressed as several forms in erythropoietic progenitor cells, including a full-length form (F-EpoR), a truncated form (T-EpoR), and a soluble form (S-EpoR). T-EpoR and S-EpoR contain the extracellular EPO-binding domain, but alternative splicing of these transcripts truncates the cytoplasmic or transmembrane domains 5.

Recombinant human erythropoietin (rHuEPO) has been widely used in tumor patients with anemia, especially those receiving chemotherapy and radiotherapy. Anemia usually impairs the life quality of tumor patients and can reduce cancer treatment effects. rHuEPO has proven effective in improving the life quality of tumor patients with anemia and reducing the requirements for blood transfusion in these patients. However, several clinical trials have shown that erythropoiesis-stimulating agents (ESAs) reduce both progression-free survival and overall survival. The use of rHuEPO is also reported to increase the risk of thrombosis in tumor patients 6-13. However, there are opposing opinions that the use of rHuEPO does not have an adverse impact on survival in tumor patients 14, 15.

In the past two decades, high levels of expression of EPO-R have been detected not only in erythroid progenitor cells, but also in many tumor cells and tissues. Some researchers hold the view that rHuEPO promotes the proliferation of 16, 17 and has anti-apoptotic effects on tumor cells 18. Furthermore, the expression levels of EPO and EPO-R in tumor tissues have been proposed as possible prognostic and predictive molecular indicators in tumor patients. It remains controversial whether rHuEPO affects the growth, proliferation, and invasion of tumor cells via the EPO-R pathway and whether EPO-R can serve as a prognostic indicator in cancer patients.

## Materials and Methods

### Cell culture and compounds

All lung cancer cell lines and MRC-5 fibroblasts were provided by the Medical Clinic V Laboratory of Ludwig-Maximilians University, Germany. HUVECs (Human Umbilical Vein Endothelial Cells) came with the Angiogenesis Starter Kit and were cultured in large vessel endothelial supplement (LVES) Medium 200 (M-200-500, Invitrogen) at 37°C in a humidified atmosphere of 5% CO_2_. The lung cancer cell lines H838 (EGFR wild type), H1975 (EGFR L858R/T790M), H1650 (EGFR exon 19 deletion), H1339 (small cell lung cancer), HCC827 (EGFR 19DelE746-A750), and MRC-5 fibroblasts were cultured in T-75 flasks (Corning®, catalogue #430641) in ATCC-formulated RPMI-1640 (ATCC 30-2001) medium and Eagle's Minimum Essential Medium, (Catalogue No.30-2003) respectively, supplemented with 10% fetal bovine serum, 100 U/mL penicillin, 100 µg/mL streptomycin, and 2 mM L-glutamine at 37°C under 5% CO_2_.

Afitinib (GIOTRIF®, Boehringer Ingelheim) and rHuEPO (500 UI, 0.3 ml, NeoRecormon®, Roche) were purchased from Germany.

### ELISAs

#### Protein extraction

The lung cancer cell lines H838, H1975, H1650, H1339, and HCC827 were cultured to approximately 85% confluence on Corning® cell culture flasks with a surface area of 75 cm^2^ and harvested separately. Cells were counted. Cells were washed two times with cold phosphate buffer saline (PBS), making sure to remove any remaining PBS after the second rinse. Then, we added 1 mL cold RIPA Buffer (Product No. 89900, Invitrogen, Darmstadt, Germany) for every 5 × 10^6^ cells and kept on ice for 5 minutes. We added a protease inhibitor cocktail (Product No. 78410, 78420 Invitrogen, Darmstadt, Germany) to the RIPA Buffer immediately before use. Protein was extracted from the monolayer-cultured mammalian cell lines as indicated by the manufacturer.

Protein concentrations were quantified using a total BCA Protein Assay Kit (Product No. 23225, Invitrogen). The cell lysates were saved and used for downstream ELISAs and Western blotting. Cell culture supernatants were also collected at different time points and stored at -80°C.

A human total Erythropoietin Receptor ELISA Kit (DYC963-5) and human VEGF DuoSet ELISA Kit (R&D Systems GmbH. Wiesbaden, Germany) were used to measure the levels of EPO-R in the cell lysates and VEGF in cell culture supernatants, according to the manufacturer's instructions.

### Western blotting

A mouse anti-EPO-R antibody was used (EpoR (D-5): sc-365662, Santa Cruz Biotechnology, Heidelberg, Germany) with a goat anti-mouse secondary IgG-HRP antibody (sc-2005, Santa Cruz). 40 μg protein from each sample was diluted with sample buffer, while ddH_2_O was added to a final volume of 35 μl. A Jurkat whole cell lysate (sc-2204, Santa Cruz Biotechnology, Heidelberg, Germany) was used as the positive control. The MagicMark™ XP Western protein standard (Invitrogen) was used to determine band sizes. The membranes were blocked in blocking buffer at room temperature for at least 1 h and afterward incubated with primary antibody overnight at 4°C. Primary antibodies were diluted 1:200 in blocking buffer. The membranes were incubated with diluted 1:5000 HRP-conjugated secondary antibodies for 1 h at room temperature the following day. Images were analyzed using image reader LAS-R software (Leica Microsystems, Germany). Integrated optical density (IOD) values were generated/analyzed with Gel-pro analyzer software (Media Cybernetics USA).

### Real-time cell proliferation profiling

Real time proliferation profiling of cell lines H1975, H1650, H838, HCC827, and HUVECs was performed using a Real-Time Cellular Analyzer (RTCA) (*i*CELLigence System, Roche Applied Science, Mannheim, Germany). An E-plate that contains interdigitated micro-electrodes integrated on its bottom was utilized for the RTCA system, which allows for label-free and real-time monitoring of cellular processes during proliferation following treatment. The electrodes will affect the local ionic environment, leading to an increase in the electrode impedance, which is represented as the Cell Index (CI). Cell impedance was measured from each individual well of the E-plate and was automatically converted to cell index values by the RTCA Software. The cell index value represents a quantitative measure of the growth status of the tested cells.

For each experiment,100 µL of complete medium was added to each well for background measurement. A cell suspension (200 μL) at a cell density of 5 × 10^3^ cells/well was seeded to each well of the E-plate (E-Plate L8 PET, Aceabio, China). The E-plate was incubated at room temperature for 30 min and placed on the reader in the incubator for continuous recording of impedance to determine the cell index (CI). After 24 h, the cells were treated with their respective compounds. The proliferation of the cells was monitored every 30 min. Each cell line was analyzed in two independent experiments. The impedance-based CI was quantified by RTCA software program version 1.2.1 (Roche, Basel. Switzerland). The data were analyzed with GraphPad Prism 5 (GraphPad Software, La Jolla, CA, USA).

### *In vitro* tube formation assay

An angiogenesis Starter Kit (Product No, A1460901, Invitrogen) was used to assess the angiogenic properties of rHuEPO in HUVECs *in vitro* after rHuEPO (50 UI/ml) treatment (versus PBS 100 µl treatment). HUVECs were seeded in LDEV-free reduced growth factor Matrigel-coated 24 well plates and incubated for 17 h at 37°C in a humidified atmosphere of 5% CO_2_.

### *In vivo* tube formation assay (Matrigel plug in nude mice)

We utilized a Matrigel plug assay to detect the angiogenic effects of rHuEPO *in vivo*, which allows for determination of the impact of various factors on the formation of functional blood vessels in mice. Five-week-old BALB/c nude mice were housed under pathogen-free conditions at the animal care vivarium of East China Normal University and treated humanely in accordance with institutional guidelines. Twenty-four nude mice were enrolled randomly in each Matrigel group: 0.6 µl Matrigel, Matrigel containing 2 × 10^6^ H838 cells, and Matrigel containing 2 × 10^6^ H1975 cells. Matrigel was injected subcutaneously into the midventral abdominal region of nude mice in a 4℃ cold room. In each group, 8 mice were randomly assigned to each of the following treatment groups: VEGF (100 ng/ml), rHuEPO (30 IU), and PBS (100 µl). Mice were treated every other day for 16 days. Afterwards, the Matrigel plugs were collected and separated from the abdominal muscle, washed with PBS, fixed in 10% formalin, and embedded in paraffin. Later immunohistochemistry (IHC) staining with an endothelial marker was used as an indicator of the presence of the newly formed capillaries in the sectioned gel plugs.

### Immunohistochemistry

Paraffin was initially removed by immersing the slides three times in xylene for 10 min. Slides were then subsequently immersed in ethanol for 5 min, progressively decreasing the percentage of ethanol (95, 85, and 75%). Slides were then rinsed in cold water before antigen retrieval, and peroxidase activity was inhibited by incubating the slides in 3% cold H_2_O_2_ for 10 min, while protected from light. The Matrigel sections were first blocked and then stained with specific endothelial marker CD31 (anti-CD31, ab124432, Abcam) and counterstained with haematoxylin. The imaging was performed using a Zeiss Axiovert 40 phase contrast microscope. Positive CD31 expression was indicative of vascular endothelial cells. Microvessel density (MVD) was determined by the Weidner method. First, the entire section was viewed at low power (× 100), after which the numbers of blood vessels in 5 randomly selected visual fields were counted under high power (× 200). The average number of vessels/field was considered the MVD of the specimen.

### Transwell migration assays

Lung cancer H838 cells were cultured to approximately 80% confluence on tissue culture plates and harvested. 2×10^5^ cells were seeded in the top chamber of the Transwell insert containing the non-coated membrane (24 well Transwell permeable support with 8 µm pores, Corning, Wiesbaden, Germany). The top chamber contained serum-free cell culture medium, and the bottom chamber contained serum-free cell culture medium with rHuEPO (50 UI/ml), VEGF (100 ng/ml), or PBS (100 µl) as the chemoattractant. The plates were incubated for 24 h, and cells remaining on the top of the membrane were removed by a cotton swab. Migration was assessed using DAPI (Invitrogen). DAPI stock solution was diluted to 300 nM in PBS and applied so that it was completely covering the membrane, which was then incubated for 1-5 minutes and rinsed three times in PBS for 10 min. Cells on the underside of the membrane were then imaged using a fluorescence microscope in at least three randomly selected fields.

### 3D collagen gel co-culture model

Before preparation of the collagen gel, cells were thawed and diluted to 2 × 10^5^ cells per well. To prepare the gels, collagen (Invitrogen), sterile 10X phosphate buffered saline (PBS), sterile distilled water (ddH_2_O), and sterile 1N NaOH were mixed on ice. The total volume of collagen gel was calculated as follows:


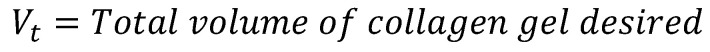
(1)


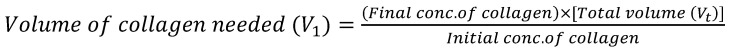
(2)


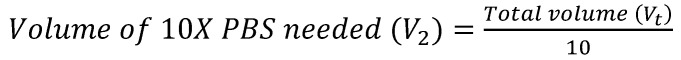
(3)


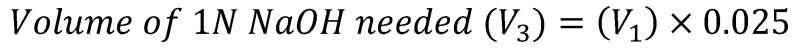
(4)


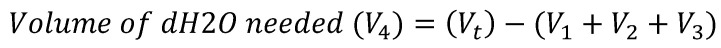
(5)

The final concentration of collagen was 1.5 mg/ml, and 0.5 ml was dispensed into each culture dish compartment and immediately placed on ice. The cells were then seeded into the collagen gel, which was pipetted several times to mix. The gels were placed at room temperature, where they solidified rapidly. Cultures were then incubated at 37°C in a humidified incubator for 30-40 minutes or until a firm gel was formed. Each dish compartment then received a total volume of 1.0 ml culture media. The cell lines H838 and MRC-5 were treated separately with rHuEPO (50 UI/ml) or PBS (100 µl) every other day.

### Statistics

All analyses were performed with SPSS 22.0 statistical software (Softonic, San Francisco, CA, USA). The data were expressed as the means ± standard deviation (S.D.) of values obtained in independent experiments. Differences between two groups were compared via a t-test. Comparisons among the three groups were performed using an ANOVA test for parametric data with a normal distribution, and the Student-Newman-Keuls test was employed as a post hoc analysis. A *p*-value < 0.05 was considered statistically significant, and a *p*-value < 0.01 was considered very significant.

## Results

### Expression of EPO-R in lung cancer cell lines

**Figure 1:**
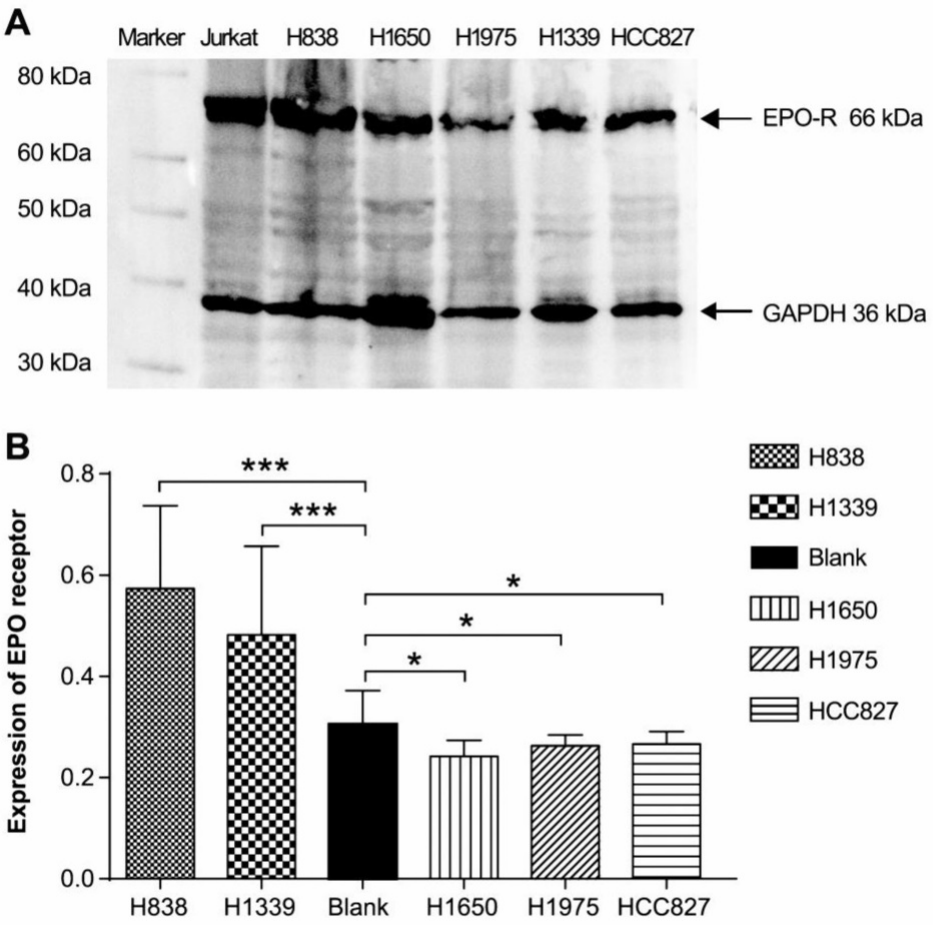
** The expression of EPO-R in lung cancer cell lines by using ELISA and Western blot**. (**A**) All cell lines (H838, H1650, H1975, H1339, and HCC827) expressed a protein of 66 kDa in size, consistent with the positive control Jurkat whole cell lysate. (**B**) In ELISA assays, only H838 and H1339 showed positive expression (n > 3, *P < 0.05, ***P < 0.001).

Using ELISAs, we determined that the EGFR wild type cell line H838 and small cell lung cancer cell line H1339 expressed EPO-R, while the EGFR gene mutation cells lines (H1975, HCC827, and H1650) did not have expression detectable above the control (Figure 1B). However, Western blotting revealed a 66 kDa band for all cell lines (Figure 1A).

### Cell proliferation in the presence of rHuEPO

**Figure 2:**
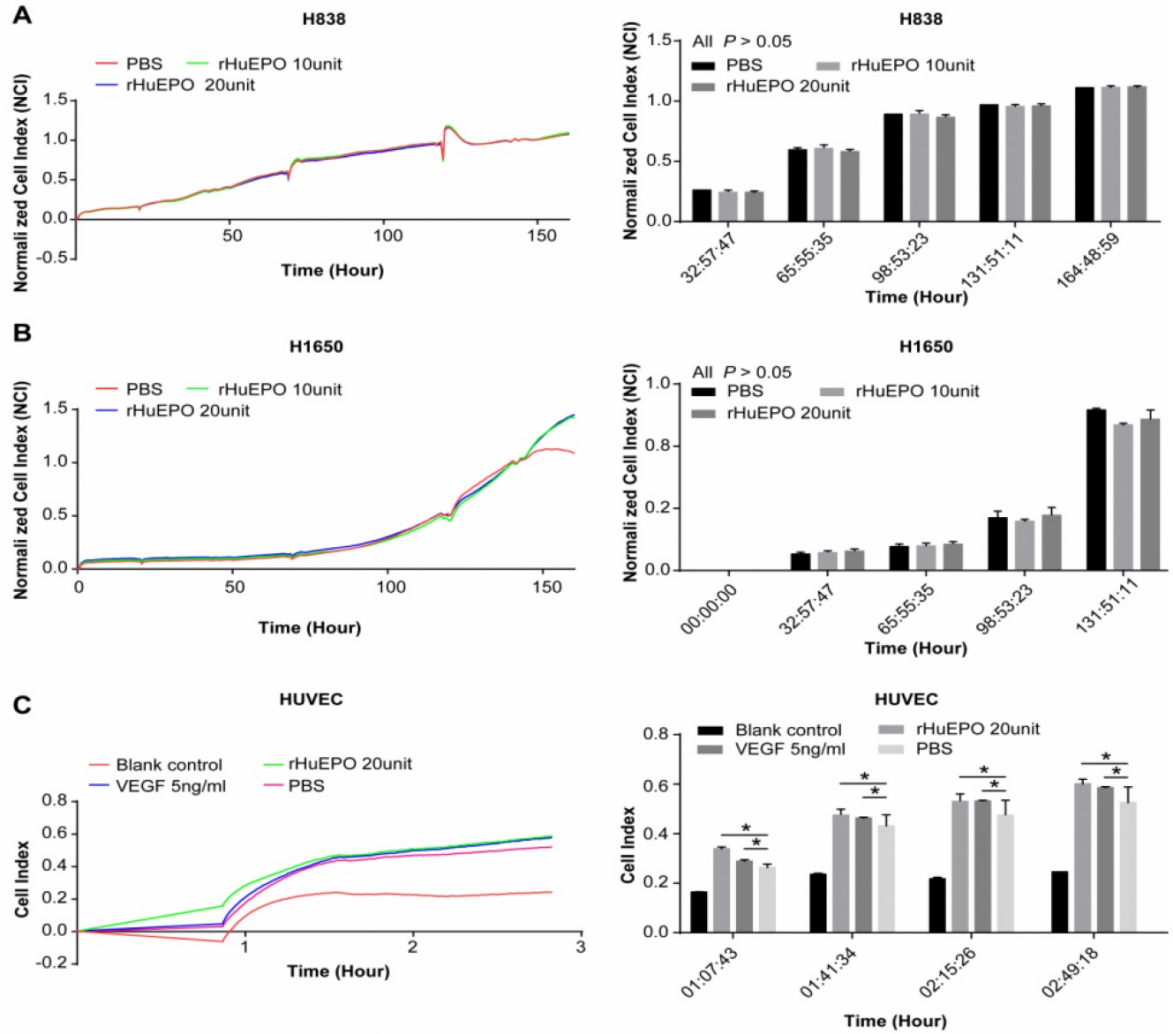
** Cell proliferation in the presence of rHuEPO.** H838 (**A**) and H1650 (**B**) were incubated in rHuEPO 10UI (green line), 20UI (blue line), or PBS (red line). In the H838 and H1650 group, there were no statistically significant differences among cells treated with rHuEPO 10 UI, 20 UI, or PBS (n = 4, *P >* 0.05) during 132 h. (**C**) HUVEC cells were incubated in rHuEPO (green line, 20 UI), VEGF (blue line 5 ng/ml), PBS (pink line), or blank control (red line) and were monitored in real-time for 3 h. The statistical analysis showed rHuEPO vs. PBS: * *P <* 0.05, VEGF vs. PBS: *P < 0.05, n = 4.

To determine whether rHuEPO promotes the proliferation of lung cancer cells and vascular endothelial cells, a Real-Time Cellular Analyzer was employed, and the lung cancer cell lines H838 and H1650 and a human vascular endothelial cell line (HUVEC) were treated with rHuEPO. Lung cancer cell lines H838 (Figure 2A) and H1650 (Figure 2B) were incubated in rHuEPO (10UI and 20UI) or PBS separately and were monitored in real-time for 165 h. HUVEC cells were incubated in rHuEPO, VEGF, or PBS and monitored in real-time for 3 h (Figure 2C). The images showed rHuEPO did not promote H838 or H1650 cell proliferation *in vitro* compared with PBS during 0-132 h, and both rHuEPO and VEGF promoted HUVEC cell proliferation compared with PBS after 2 h measured by the iCELLigence System.

### rHuEPO induces HUVEC tube formation

**Figure 3:**
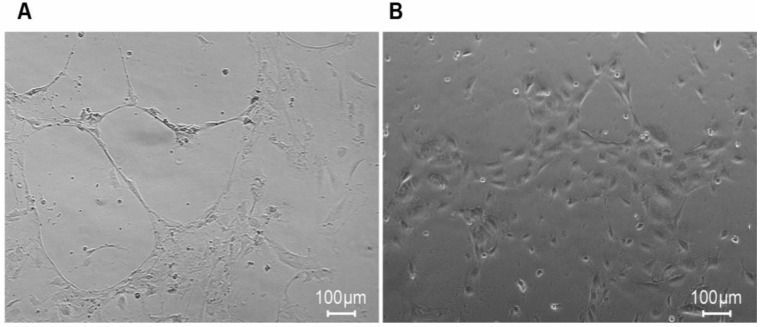
** rHuEPO induces HUVEC tube formation.** (**A**) HUVECs were incubated in rHuEPO after 17 h on Matrigel coated 24 well plates. (**B**) HUVECs were incubated in PBS after 17 h on Matrigel coated 24 well plate. The images showed rHuEPO strongly promoted HUVEC tube formation *in vitro.*

We found that rHuEPO and VEGF promoted the proliferation of HUVECs *in vitro*. Thus, we performed a HUVEC tube formation assay in the presence of rHuEPO versus PBS to assess the angiogenic properties of rHuEPO *in vitro*. In the microscopy images, HUVEC tube formation in the presence of rHuEPO appeared as organized capillary-like structures (Figure 3A), while the PBS treatment showed scattered cells (Figure 3B).

### rHuEPO strongly stimulates angiogenesis *in vivo*

**Figure 4:**
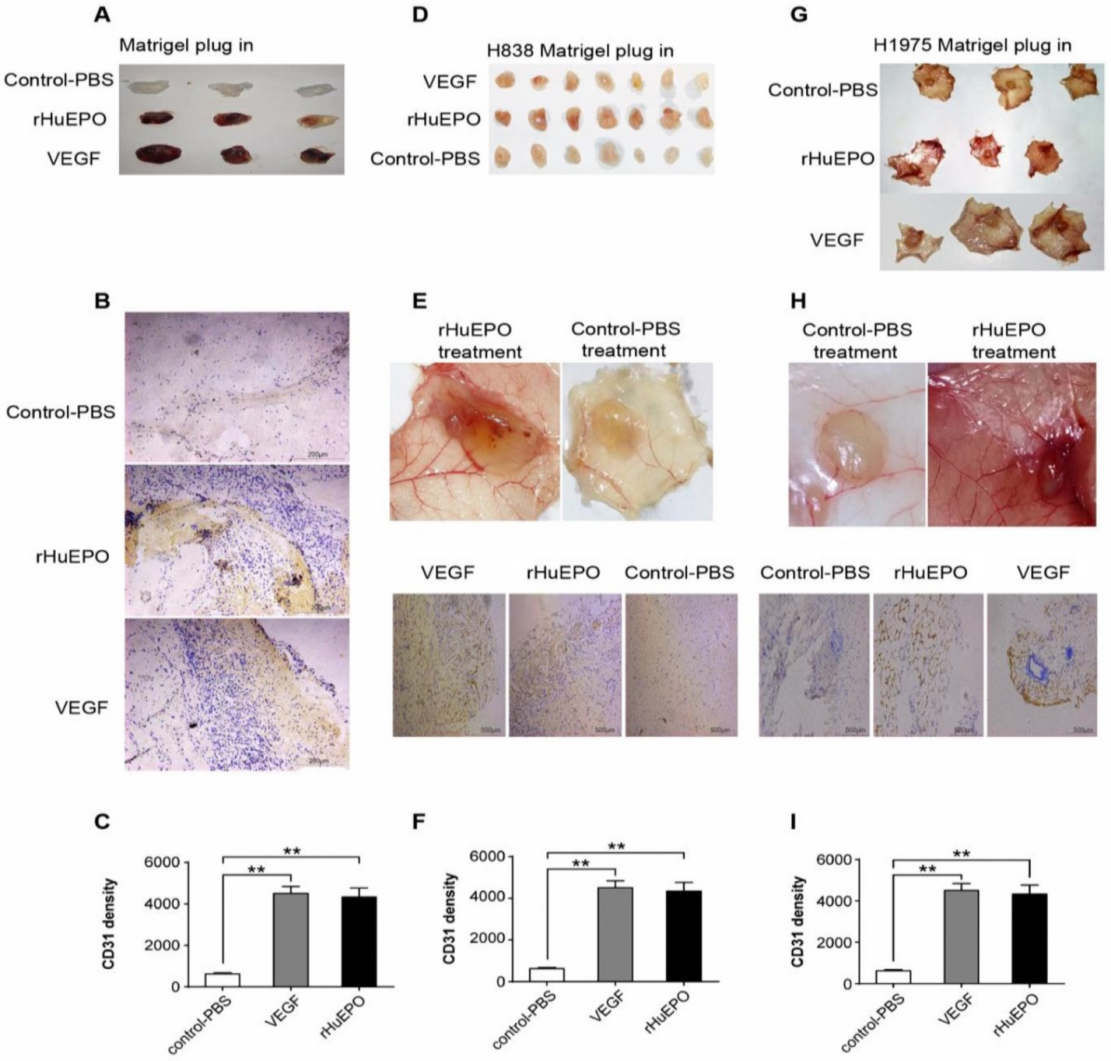
** rHuEPO strongly stimulates angiogenesis *in vivo*.** (**A**) BALB/c mice were injected subcutaneously with 0.6 µl of Matrigel. (**D**) Matrigel containing H838 cells, or (**G**) Matrigel containing H1975 cells. (**B, E, H**) CD31 expression was used to assess vessel density and is represented as MVD. (**C, F, I**). N = 8. **P < 0.05 compared with PBS control group.

Because rHuEPO stimulated angiogenesis *in vitro* we used a Matrigel plug to assess its angiogenic properties *in vivo* in nude mice. Eight mice in each group received rHuEPO (30UI), VEGF (100 ng/ml), or PBS (100 µl) treatment every other day. After 16 days, the mice were euthanized, and the Matrigel plugs were excised, photographed, and stained by CD31 immunohistochemisty (Figure 4). We found that rHuEPO has strong angiogenic properties *in vivo*.

### rHuEPO does not influence H838 lung cancer cell migration

**Figure 5:**
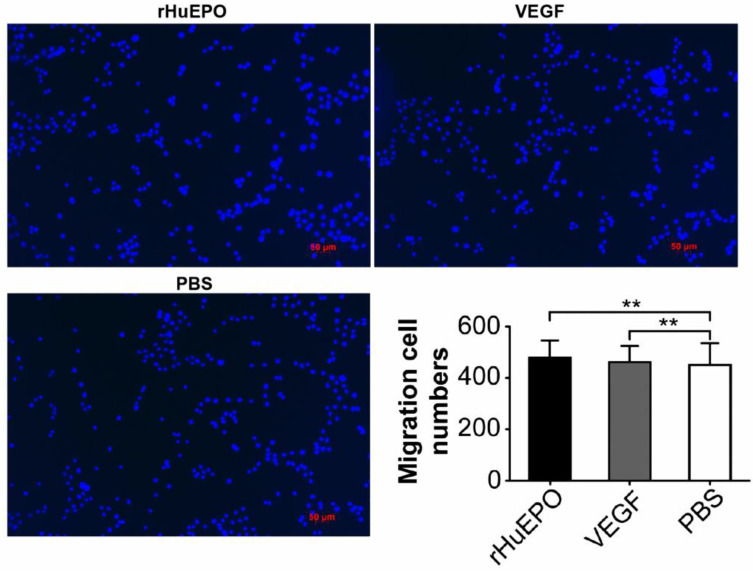
** H838 transwell migration assays in the presence of rHuEPO, VEGF, and PBS.** Transwell assays were used to evaluate the effects of rHuEPO and VEGF versus PBS. Cells were stained with DAPI (blue). **P > 0.05 for rHuEPO vs. PBS, VEGF vs. PBS.

Lung cancer cell line H838 transwell migration assays were performed in the presence of rHuEPO, VEGF, or PBS to determine if rHuEPO influences lung cancer cell migration (Figure 5). However, we found that rHuEPO does not induce H838 cell migration.

### rHuEPO does not antagonize the effect of Afatinib *in vitro*

**Figure 6:**
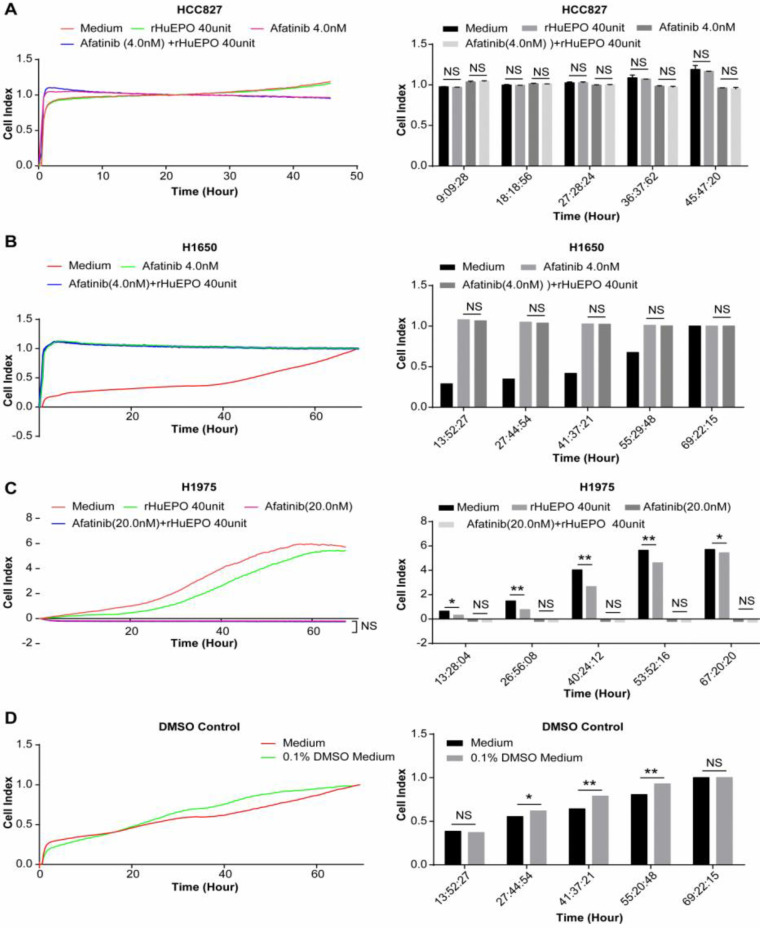
** Simultaneous treatment with rHuEPO and EGFR-TKI (Afatinib) in lung cancer cell lines.** (**A**) The blue and the pink lines in the HCC827 group indicate rHuEPO (40 UI) + Afatinib (4 nM) and Afatinib (4 nM) treatment, respectively, and (**C**) in the H1975 group rHuEPO (40UI) + Afatinib (20 nM) and Afatinib (20 nM), respectively. (**B**) In the cell line H1650, the green line represents Afatinib (4 nM), and blue line represents rHuEPO (40 UI) + Afatinib (4 nM). The lines in both the rHuEPO + Afatinib and Afatinib groups were nearly coincident, and there were no statistically significant differences between the rHuEPO + Afatinib and the Afatinib only treatment group (n = 4, *P >* 0.05).

We monitored the proliferation of EGFR mutation lung cancer cell lines H1650, H1975, and HCC827 using a Real-Time Cellular Analyzer (RTCA) under simultaneous treatment with rHuEPO and Afatinib, which we compared to Afatinib treatment alone. Lung cancer cell lines HCC827 and H1650 were incubated in rHuEPO (40UI) and Afatinib (4 nM) or Afatinib (4 nM) alone (Figure 6A,B). The cell line H1975 was incubated in rHuEPO (40 UI) and Afatinib (20 nM) or Afatinib (20 nM) alone and monitored in real time by the iCELLigence System (Figure 6C). We found that rHuEPO did not antagonize the inhibitory effects of Afatinib. DMSO was used to suspend the Afatinib; 0.1% DMSO itself did not reduce cell proliferation (Figure 6D).

### The expression of VEGF in a 3D cell culture model in the presence of rHuEPO or PBS

**Figure 7:**
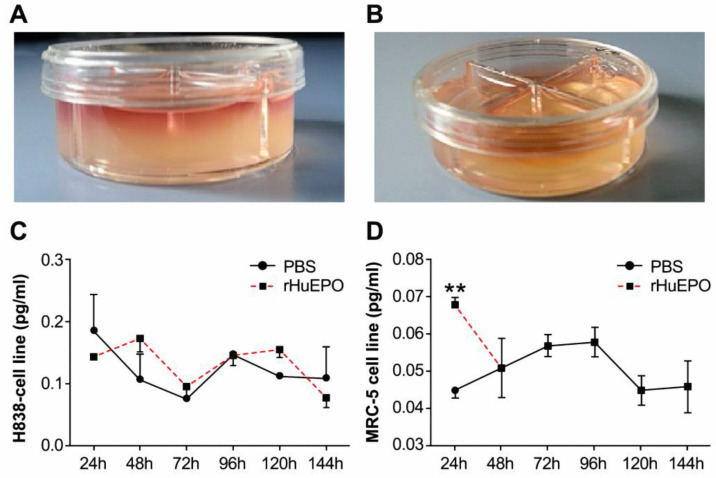
** 3D collagen co-culture model.** (A) 1.0 ml cell culture medium permeated into the collagen gel from the top side, and the cultivated cells were suspended in the 3D collagen gel below. (B) 0.5 ml collagen gel was allowed to solidify in every culture dish compartment independently. The graphs depict VEGF expression in H838 (**C**) and MRC-5 (**D**) cells in mono-cultures treated with rHuEPO or PBS. There were no statistically significant differences between the rHuEPO and PBS treatment groups in the H838 or MRC-5 mono-culture groups (n = 6, *P >* 0.05).

We established a 3D cell culture model (Figure 7A,B). The two cell lines MRC-5 (Figure 7D) and H838 (Figure 7C) were treated with 50 UI/ml rHuEPO or 100 µl PBS every other day, and the cell culture supernatants were collected at six time points for ELISA. We found rHuEPO treatment did not change VEGF expression versus PBS control.

## Discussion

Although rHuEPO has been widely used in tumor-associated anemia, the expression of EPO-R in tumor cells, the working mechanism of EPO and EPO-R, and the results of clinical trials associated with rHuEPO remain controversial. As shown by an accumulating body of evidence 16, 19-23, EPO-R is highly expressed in many tumor cell types, in addition to erythroid progenitor cells. In this study, we analyzed the expression of EPO-R in lung cancer cell lines H838, H1975, H1650, H1339, and HCC827 using the most frequently used commercially available ELISA kit and Western blotting antibody. We found a discrepancy in EPO-R expression in lung cancer cells using these two methods. We detected positive expression in only two cell lines via ELISA, while we detected positive expression in all cell lines by Western blot. This finding raised the question as to whether the commercially available EPO-R kits or antibodies for detecting EPO-R are specific. We conducted a search of the commercial EPO-R antibodies directed at detecting EPO-R. The predicted sizes of EPO-R using commercial anti-EPO-R antibodies ranged from 50 to approximately 64-78 kDa via Western blotting. The research results obtained using products from different manufacturers could influence the overall results, which may not agree between such studies. Moreover, the calculated size of the EPO-R protein is 52.6 kD, and maturation with the addition of a carbohydrate can increase the size to approximately 57 kD. However, when a tagged EPO-R was over-expressed, antibodies against the protein tag and EPO-R identified an EPO-R that was around 59 kDa 24. Elliott et al. used a polyclonal antibody, C-20, to detect EPO-R in cell lines from breast, brain, cervix, and kidney tissue. The detected 66-kD band, which investigators believed to be EPO-R, was then checked by sequence analysis. The results showed that the 66 kDa band protein did not correspond to EPO-R, rather to heat shock proteins (HSPs), including HSP70-2 and HSP70-5 24. Our study also showed that all tested lung cancer cell lines were positive for a 66 kDa protein. Given a lack of specific commercially available EPO-R antibodies, the positive expression of EPO-R, as detected in our study, remains uncertain. Moreover, some reports failed to clarify the type of EPO-R that they detected. Therefore, conclusions from studies using these commercially available antibodies, which stated that EPO-R was highly expressed in tumor tissues and cells and could serve as a prognostic indicator for cancer patients, should be reexamined.

Next, we used reliable real-time cell proliferation monitoring technology to monitor the proliferation of the EGFR-wild type lung cancer cell line H838 (both ELISA and Western blot results indicated positive EPO-R expression) and the EGFR mutation cell line H1650 (only positive via Western blot) in the presence of rHuEPO or physiological buffer control (PBS). Two dose groups were established, and the drug was given once every two days until 165 h. Both doses of rHuEPO failed to promote *in vitro* proliferation of the two lung cancer cell lines tested. We also monitored the proliferation of EGFR mutation lung cancer cells H1650, H1975, and HCC827 under treatment with rHuEPO and Afatinib versus Afatinib alone. Our data showed that during a course of approximately 60 h, rHuEPO did not antagonize the inhibitory effects of Afatinib. Therefore, it is likely that functional EPO-R was not expressed in these lung cancer cell lines, despite the Western blotting results. In the absence of functional EPO-R, the downstream cellular signaling pathway was not activated, despite the fact that the administered dose of rHuEPO far exceeded the usual dose used in humans. Therefore, it is possible that (1) EPO-R is absent or non-functional in lung cancer cells, (2) EPO-R is not expressed in the membrane of lung cancer cells or very little EPO-R is expressed, or (3) EPO has very low affinity for EPO-R in cancer cells.

Some studies have detected EPO-R expression at the mRNA level. Frille A et al. 25 reported 3 lung cancer cell lines expressed EPO-R at the mRNA and protein level in both normoxic and hypoxic conditions, but no erythropoietin-induced growth was observed in non-small cell lung cancer cells. This finding is in agreement with our results. Furthermore, Fecková B et al. 26 also determined that CpG methylation in exon 1 did not play a significant role in the regulation of EPO-R transcription in selected human cancer cells. We compared the transcriptional levels of EPO-R in a total of 18 patients, comparing lung cancer tissue to tumor-free tissue from the same patient (control). EPO-R was expressed in both cancerous and normal lung tissues, but there were no statistically significant differences between the two, as shown in the supplemental data (Table S1,S2). Understandably, there are many processes that occur between transcription and translation, and the relationship between mRNA and protein is not always one for one. In this regard, we believe that detecting mRNA expression to help examine the expression this receptor protein in lung cancer is inappropriate in our study.

To determine whether rHuEPO induces the migration of tumor cells, we examined the effects of VEGF, rHuEPO, and PBS on the migration capacity of H838 cells. The results showed rHuEPO and VEGF did not induce the migration of tumor cells any better than the PBS control. We also analyzed whether rHuEPO was capable inducing tumor and tumor stromal cells to secret VEGF, which could indirectly promote angiogenesis. We established a 3D cell culture model with H838 or human lung fibroblast cells (MRC-5) 27. We found that rHuEPO treatment did not change VEGF expression (versus PBS control), although Batra et al.^28^ previously reported that exogenous erythropoietin increased the production and secretion of angiogenic growth factors, vascular endothelial growth factor, and placenta growth factor from tumor cell lines.

Interestingly, although rHuEPO did not promote the proliferation of lung cancer cells *in vitro*, it significantly enhanced the proliferation of vascular endothelial cells (HUVECs). We found that after treatment with rHuEPO (20UI) or VEGF (5ng/ml) for 2h, HUVECs displayed enhanced proliferation. This was in agreement with previously published studies 29-31. We can conclude from these results that (1) vascular endothelial cells may share common receptors with erythroid progenitors cells, so that rHuEPO can activate downstream HUVEC signaling pathways to enhance proliferation, and (2) rHuEPO may promote vascular endothelial cell tube formation and therefore have an angiogenic effect *in vivo.*

The subsequent HUVEC tube formation assays demonstrated the effects of rHuEPO on HUVEC tube formation *in vitro*. Accordingly, a Matrigel plug assay was performed to assess the angiogenic properties of rHuEPO *in vivo*. The Matrigel plug angiogenesis assay is a simple *in vivo* technique to detect newly formed blood vessels in the transplanted gel plugs in nude mice, which removes the complicating factors present within tumor tissues that may skew results. We found that compared to the PBS control, Matrigel alone (no cells), Matrigel containing H838 cells, and Matrigel containing H1975 cells all displayed a significant angiogenic effect in the presence of rHuEPO or VEGF. Many previous studies have confirmed the angiogenic effects of rHuEPO *in vitro*, while our results further indicate that rHuEPO has strong angiogenic effects *in vivo*.

Furthermore, microthrombosis were observed in some Matrigel plugs in the rHuEPO treatment group. rHuEPO promotes the production of red cells. This leads to the changes in the local hemodynamics, while the neovascular structure in cancer is poorly formatted. Therefore, it could easily trigger a downstream coagulation cascade reaction. In addition, it could increase the likelihood of tumor embolism and metastasis through the creation of new blood vessels*.*

This helps to explain the observation in previous clinical trials that rHuEPO increased the risk of thrombosis in tumor patients. As a result, some patients suffered myocardial infarction, stroke, venous thromboembolism, thrombosis of vascular access, or even death after rHuEPO treatment. The risk is even higher in tumor patients already carrying risk factors for thrombosis, such as a history of thromboembolism, recent surgery, hormonal agents, hypercoagulability, elevated pre-chemotherapy platelet counts, hypertension, steroids, and prolonged immobilization 6-13.

In 2017, the U.S. Food &Drug Administration determined that the ESA Risk Evaluation and Mitigation Strategy (REMS), which was limited to the use of rHuEPO to treat patients with anemia due to associated myelosuppressive chemotherapy is no longer necessary to ensure that the benefits of rHuEPO outweigh its risks of shortened overall survival and/or increased risk of tumor progression or recurrence in patients with cancer 32. However, in the latest NCCN guidelines, it is still emphasized that rHuEPO should be used, but only in strict accordance with the guidelines and based on a full consideration of the above risks.

We found that rHuEPO did not promote the proliferation and migration of lung cancer cells, nor did it antagonize apoptosis caused by the molecular targeted drug Afatinib. However, in previous studies, some tumor patients showed an increased risk of shortened overall survival and/or an increased risk of tumor progression or recurrence after rHuEPO treatment. Other reasons for this observation, as demonstrated in our study, include an rHuEPO-increased risk of thrombosis *in vivo*. rHuEPO may have a close relationship to tumor cell metastasis to the peripheral blood through the formulation of new blood vessels and may prompt tumor stromal cells to secret cytokines that promote tumor cell migration and invasion, as implied by Ribatti et al. Human EA.hy926 endothelial cells express an EPO receptor that binds to JAK2 and induces its transient activation after rHuEPO exposure. Furthermore, rHuEPO exposure resulted in a three-fold greater matrix metalloproteinase 2 (MMP-2) activity in rHuEPO-treated EA.hy926 cells compared to untreated cells 31.

In summary, our present study demonstrates that erythropoietin promotes blood vessel growth (angiogenesis) *in vitro* and *in vivo*, which can increase the risk of a potentially serious complication (thrombosis) that can lead to cardiovascular disease and even death, in addition to supporting tumor growth and spread. Furthermore, it raises some concerns over published results on EPO-R antibodies, which our findings suggest may not recognize EPO-R specifically, thus leading to inappropriate conclusions.

## Supplementary Material

Supplementary table S1.

Supplementary table S2.

## Abbreviations

rHuEPO: Recombinant human erythropoietin
ELISA: Enzyme linked immunosorbent assay
PBS: Phosphate buffer saline
HUVECs: Human umbilical vein endothelial cells
VEGF: Vascular endothelial growth factor
MVD: Microvessel density
EPO-R: EPO-receptor
MRC-5: Human lung fibroblast cells
IHC: Immunohistochemistry
EGFR-TKI: Epidermal growth factor receptor-tyrosine kinase inhibitor
RTCA: Real-Time Cellular Analyzer
CI: Cell Index
HSPs: Heat shock proteins
ESAs: Erythropoiesis-stimulating agents
REMS: Risk Evaluation and Mitigation Strategy
