# Supplementary material for: Role of the erythropoietin receptor in Lung Cancer cells: erythropoietin exhibits angiogenic potential
Source: J Cancer. 2020 Aug 21;11(20):6090–100. doi: 10.7150/jca.36924 (PMC7477424; doi:10.7150/jca.36924)
Supplement: Supplementary file 1 — Supplementary table S1. [file jcav11p6090s1.pdf]

Table S 1. Comparison between LU and TU

group	N	Mean	Standard deviation	*t*	P
LU	18	284.2725	83.50767	1.934	0.064
TU	18	201.0509	162.38801		
